# GLP-1 Receptor Agonists and Myocardial Perfusion: Bridging Mechanisms to Clinical Outcomes

**DOI:** 10.3390/ijms26073050

**Published:** 2025-03-26

**Authors:** Paschalis Karakasis, Dimitrios Patoulias, Panagiotis Theofilis, Konstantinos Pamporis, Marios Sagris, Panayotis K. Vlachakis, Theocharis Koufakis, Antonios P. Antoniadis, Nikolaos Fragakis

**Affiliations:** 1 Second Department of Cardiology, Hippokration General Hospital, Aristotle University of Thessaloniki, Konstantinoupoleos 49, 54642 Thessaloniki, Greece; aantoniadis@gmail.com (A.P.A.); fragakis.nikos@googlemail.com (N.F.); 2 Second Propedeutic Department of Internal Medicine, Faculty of Medicine, School of Health Sciences Aristotle, University of Thessaloniki, 54124 Thessaloniki, Greece; thkoyfak@auth.gr; 3 First Cardiology Department, School of Medicine, Hippokration General Hospital, National and Kapodistrian University of Athens, 15772 Athens, Greece; panos.theofilis@hotmail.com (P.T.); konstantinospab@gmail.com (K.P.); masagris1919@gmail.com (M.S.); vlachakispanag@gmail.com (P.K.V.)

**Keywords:** glucagon-like peptide-1 receptor agonists, GLP-1RAs, myocardial perfusion, microvascular dysfunction, diabetes, obesity

## Abstract

Coronary microvascular dysfunction (CMD) is a key contributor to myocardial ischemia and adverse cardiovascular outcomes, particularly in individuals with metabolic disorders such as type 2 diabetes (T2D). While conventional therapies primarily target epicardial coronary disease, effective treatments for CMD remain limited. Glucagon-like peptide-1 receptor (GLP-1R) agonists have emerged as promising agents with cardiovascular benefits extending beyond glycemic control. Preclinical and clinical evidence suggests that GLP-1R activation enhances coronary microvascular function through mechanisms including improved endothelial function, increased nitric oxide bioavailability, attenuation of oxidative stress, and reduced vascular inflammation. Moreover, GLP-1R agonists have been shown to improve myocardial blood flow, myocardial perfusion reserve, and coronary endothelial function, particularly in high-risk populations. Despite these promising findings, inconsistencies remain across studies due to variability in patient populations, study designs, and imaging methodologies. This review summarizes current evidence on the role of GLP-1R agonists in myocardial perfusion, bridging mechanistic insights with clinical outcomes. Further large-scale, well-designed trials are needed to clarify their long-term impact on coronary microcirculation and explore their potential as targeted therapies for CMD.

## 1. Introduction

Coronary microvascular dysfunction (CMD) is increasingly recognized as a key contributor to myocardial ischemia, heart failure, and adverse cardiovascular outcomes, even in the absence of obstructive coronary artery disease [1]. Coronary microcirculation plays a crucial role in regulating myocardial perfusion, ensuring adequate oxygen delivery in response to dynamic metabolic demands. However, impairments in endothelial function, nitric oxide (NO) bioavailability, vascular inflammation, and oxidative stress can disrupt this finely tuned system, leading to reduced myocardial blood flow and increased cardiovascular risk [2]. Despite its clinical significance, effective targeted therapies for CMD remain limited, necessitating novel pharmacological approaches to improve coronary microvascular function [3].

Glucagon-like peptide-1 receptor (GLP-1R) agonists, initially developed for the treatment of type 2 diabetes (T2D) and obesity, have emerged as promising agents with significant cardiovascular benefits beyond glycemic control [4,5,6,7,8,9,10,11,12,13]. These agents have been shown to enhance endothelial function, improve vasodilation, reduce vascular inflammation, and modulate oxidative stress—mechanisms that may collectively contribute to improved myocardial perfusion and coronary microvascular health. Preclinical and clinical studies have demonstrated that GLP-1R activation can augment myocardial blood flow, increase microvascular blood volume, and improve myocardial perfusion reserve, particularly in patients with metabolic syndrome and diabetes, populations at heightened risk for microvascular dysfunction [14].

Despite accumulating evidence supporting the vasoprotective effects of GLP-1R agonists, inconsistencies exist across studies regarding their impact on coronary microcirculation. Variability in study design, patient populations, imaging modalities, and outcome measures has contributed to discrepancies in reported outcomes. This review aims to bridge the gap between mechanistic insights and clinical applications by summarizing the physiological effects of GLP-1R agonists on coronary microcirculation, evaluating clinical evidence supporting their role in myocardial perfusion, and discussing potential therapeutic implications for patients with CMD. Understanding the cardioprotective properties of GLP-1R agonists may pave the way for their integration into cardiovascular risk management strategies, expanding their utility beyond metabolic disease and toward targeted microvascular therapy.

## 2. Coronary Microvascular Dysfunction: Deciphering the Pathophysiology

The structural integrity of the myocardium and the optimal function of the heart are fundamentally dependent on sufficient coronary blood flow, encompassing both the major coronary arteries and the intricate microvascular network [15,16,17]. While the large coronary arteries accommodate substantial blood volumes, the coronary microcirculation, comprising vessels with internal diameters of less than 5000 μm, plays a pivotal role in dynamically regulating perfusion to meet the fluctuating oxygen demands of the myocardium [18]. Numerous cardiovascular risk factors and pathological conditions contribute to impairments in myocardial microcirculation, ultimately compromising myocardial perfusion and predisposing to ischemic injury [19,20,21]. This section provides a comprehensive overview of the key risk factors and disease states that disrupt coronary microvascular perfusion.

### 2.1. Inflammation-Driven Dysregulation

Dysfunctional microvascular endothelial activity is intricately interwoven with both systemic and localized inflammatory processes [22]. Inflammatory mediators modulate the secretion of NO and cytokines, thereby impairing the autoregulatory capacity of coronary blood flow [23]. Moreover, endothelial dysfunction within the coronary microcirculation perpetuates a cycle of inflammation and immune activation, characterized by the release of pro-inflammatory cytokines such as interleukin and tumor necrosis factor-alpha (TNF-α) [24]. Beyond its localized effects, systemic immune-inflammatory responses associated with macrovascular atherosclerosis exacerbate this dysfunction by elevating circulating levels of TNF-α and C-reactive protein, which in turn engage endothelial surface receptors within the microvasculature [25,26]. This inflammatory cascade culminates in a significant downregulation of endothelial NO synthase (eNOS) expression and NO bioavailability, ultimately disrupting vasomotor tone and impairing the adaptive capacity of the coronary microcirculation [27]. Key molecular mediators of large-vessel atherosclerosis, including Toll-like receptors (TLRs), NLRP3 inflammasome, and nuclear factor kappa B (NF-κB), are also potent inducers of oxidative stress within the microvascular endothelium [28]. This heightened oxidative burden disrupts endothelial homeostasis, impairing its regulatory capacity and predisposing the microcirculation to dysfunction.

Beyond localized vascular effects, systemic inflammatory disorders, such as chronic autoimmune diseases, provoke dysregulated immune activation, particularly involving T lymphocytes and monocytes [29,30]. This pathological immune response extends to the coronary microvasculature, where it perturbs endothelial function and contributes to maladaptive vascular remodeling. Evidence further suggests that systemic inflammation is linked to exacerbated ischemic electrocardiographic abnormalities, particularly in individuals with elevated circulating C-reactive protein (CRP) levels, underscoring the inflammatory regulation of coronary microcirculatory dynamics [31].

Metabolic dysregulation, particularly in the context of obesity, compounds these effects by fostering a pro-inflammatory milieu characterized by increased levels of leptin, adiponectin, interleukin-6 (IL-6), and TNF-α [32,33]. These adipokines and cytokines act synergistically to disrupt endothelial signaling, impair vasomotor function, and ultimately compromise the adaptive perfusion capacity of the coronary microvasculature [32,33].

### 2.2. Oxidative Stress-Induced Injury

Oxidative stress, driven by the excessive production and accumulation of reactive oxygen species (ROS), alongside the ensuing inflammatory cascade, represents a fundamental pathogenetic axis in the development of coronary microvascular dysfunction (CMD) [34]. Under hyperglycemic conditions, mitochondrial electron transport chain activity intensifies, leading to a substantial increase in superoxide anion generation [35]. This surge in oxidative stress is further amplified via the protein kinase C (PKC)/NADPH oxidase signaling axis, exacerbating intracellular ROS accumulation [35].

Beyond direct oxidative injury, mitochondrial-derived superoxide anions play a critical role in accelerating the formation of advanced glycation end products (AGEs) within endothelial cells [36]. This AGEs-mediated endothelial dysfunction further exacerbates CMD through activation of the endoplasmic reticulum (ER) stress pathway, specifically engaging the PERK/CaN/NFATc4 signaling cascade [36]. The resultant cellular stress response disrupts microvascular homeostasis, promoting maladaptive vascular remodeling and impairing coronary perfusion regulation [36].

### 2.3. Hyperglycemia and Insulin Resistance

Hyperglycemia and insulin resistance are central pathological drivers of microvascular dysfunction in diabetes, reinforcing a reciprocal cycle of metabolic and vascular impairment [2,37]. Elevated blood glucose levels disrupt endothelial homeostasis by impairing NO-dependent vasodilation and amplifying intracellular oxidative stress within microvascular endothelial cells, ultimately dysregulating NO metabolism and promoting endothelial dysfunction [2].

Conversely, microvascular dysfunction itself exacerbates diabetes progression by impairing insulin and glucose transport, further compounding metabolic dysregulation [38]. Insulin resistance contributes to this dysfunction by attenuating NO production and eNOS activity, while sustained hyperinsulinemia leads to excessive fatty acid accumulation [35,39]. This, in turn, activates PKC, triggering a pro-inflammatory cascade that further compromises microvascular integrity [35,39]. Notably, metabolic insulin resistance is closely intertwined with vascular and microvascular insulin resistance, highlighting their interconnected pathophysiology [40]. The evidence suggests that myocardial perfusion is significantly diminished in individuals with T2D, particularly in the postprandial state, emphasizing the systemic impact of microvascular dysfunction on cardiac metabolic regulation [41].

## 3. Preclinical Models and Human Studies Investigating the Effect of GLP-1 Agonism on Coronary Microvascular Perfusion

Since the identification of GLP-1, its multifaceted physiological roles have been progressively elucidated. Primarily synthesized by enteroendocrine cells, GLP-1 exerts a critical influence on systemic glucose homeostasis through its interaction with GLP-1R, which is widely expressed across various tissues, including endocrine glands and skeletal muscle [42]. Currently, selective GLP-1R agonists have been approved for clinical use in the management of T2D and obesity, owing to their potent glycemic and weight-regulatory effects [43,44,45]. Beyond their glucometabolic benefits, GLP-1R activation has been increasingly recognized for its cardioprotective properties [46,47,48,49]. Given the accumulating evidence, the subsequent discussion will explore the mechanistic pathways through which GLP-1 and its receptor agonists modulate coronary microcirculation, providing insight into their potential therapeutic implications in cardiovascular health.

### 3.1. Microvascular Dilation and Blood Pressure Lowering

**Figure 1:**
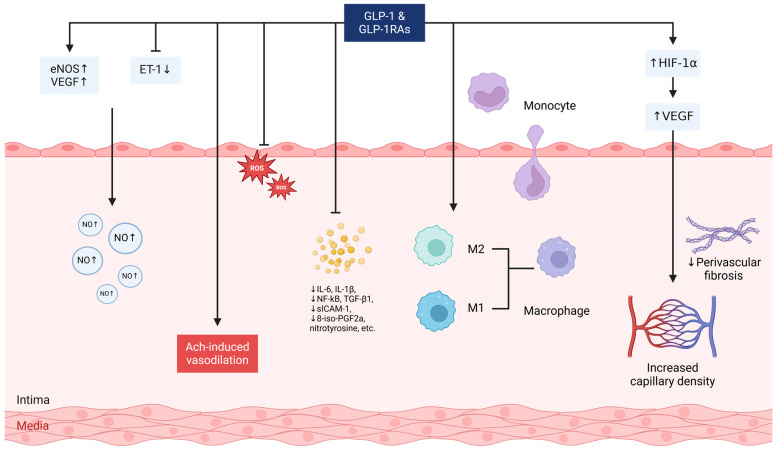
Vascular and microvascular effects of GLP-1 and GLP-1R agonists on myocardial perfusion. GLP-1 and GLP-1R agonists improve myocardial microvascular perfusion by upregulating eNOS and VEGF while reducing the vasoconstrictive factor ET-1, thereby preserving nitric oxide (NO) levels and enhancing microvascular dilation. They also augment Ach-induced vasodilation and exert endothelial protection by reducing oxidative stress and vascular inflammation. GLP-1 signaling promotes a shift from the pro-inflammatory M1 macrophage phenotype to the reparative M2 phenotype, which is associated with reduced inflammatory cytokine secretion, enhanced extracellular matrix remodeling, and improved tissue repair. This shift contributes to the resolution of inflammation, stabilization of vascular function, and maintenance of myocardial perfusion. Additionally, GLP-1 and GLP-1R agonists stimulate angiogenesis through the HIF-1α and VEGF pathways, supporting capillary formation and myocardial oxygenation. These effects collectively enhance vascular integrity and optimize coronary microcirculation. Abbreviations: eNOS, endothelial NO synthase; ET-1, endothelin-1; HIF-1α, hypoxia-inducible factor-1α; NO, nitric oxide; ROS, reactive oxygen species; VEGF, vascular endothelial growth factor.

The structural integrity and functional competence of endothelial cells are fundamental to the dynamic regulation of vascular tone and blood flow modulation [50]. Endothelial dysfunction represents an early hallmark of coronary vascular disease, often precipitated by a reduction in eNOS expression, impaired phosphorylation of eNOS at the serine 1177 residue, and diminished availability of essential eNOS substrates or cofactors [51]. Collectively, these disturbances culminate in decreased NO bioavailability, impairing vasodilatory capacity and predisposing to vascular pathology [52]. GLP-1R agonists, such as exenatide, have demonstrated the ability to enhance NO production and promote eNOS phosphorylation, thereby significantly improving coronary endothelial function. This vasoprotective effect is thought to be mediated through the activation of the AMPK/PI3K-Akt/eNOS signaling cascade via a GLP-1R/cAMP-dependent mechanism [53]. Figure 1 provides a schematic illustration summarizing the key mechanisms by which GLP-1 and GLP-1 receptor agonists improve myocardial microvascular perfusion.

Liraglutide, a GLP-1R agonist, has also been shown to enhance NO activity, promoting microvascular dilation and improving perfusion [54,55]. Of note, the activation of eNOS by GLP-1 receptor agonists appears to be predominantly endothelium-specific, as evidenced by studies showing that their vasodilatory effects are abolished in models with endothelial-specific GLP-1R deletion [55]. While low-level GLP-1R expression has been identified in other tissues, current evidence suggests that the observed vascular benefits are largely receptor-mediated and confined to the endothelium, with minimal off-target effects reported at therapeutic doses [56]. Mechanistic investigations suggest that liraglutide preserves endothelial function by preventing the decline of NO and endothelium-derived hyperpolarization factors within the microvasculature [54]. This effect is mediated through the upregulation of eNOS and VEGF expression, coupled with a reduction in the vasoconstrictor ET-1, thereby augmenting microvascular dilation and myocardial microvascular perfusion. Notably, these vasoprotective properties of liraglutide are abolished when GLP-1R is genetically deleted, either globally or selectively in endothelial cells, underscoring the receptor’s essential role in mediating its vascular benefits [55]. Similarly, GLP-1 has been shown to elevate intracellular cAMP and NO levels in human umbilical vein endothelial cells (HUVECs), reinforcing its role in preserving endothelial integrity and function [57].

In Dahl salt-sensitive rats, prolonged GLP-1 administration has been shown to enhance endothelial function and attenuate hypertension progression, likely through the augmentation of acetylcholine (ACh)-induced vasodilation [58]. This vasodilatory effect of GLP-1 has also been observed in healthy individuals, suggesting its broader physiological relevance [59]. Given the vasoprotective properties of GLP-1, GLP-1R agonists may exert similar benefits. Preclinical studies further support this notion, as liraglutide treatment of obese Zucker rats successfully restored ACh-induced microvascular dilation [54]. In individuals with newly diagnosed T2D, a six-month course of liraglutide has been associated with improved endothelial function, as evidenced by enhanced flow-mediated dilation, as well as a reduction in arterial stiffness [60]. The resultant decrease in afterload may contribute to improved myocardial perfusion, highlighting the cardiometabolic benefits of GLP-1R activation. Beyond its direct endothelial effects, emerging evidence suggests that GLP-1R activation in atrial myocytes promotes blood pressure reduction through an EPAC2-dependent mechanism, facilitating atrial natriuretic peptide (ANP) release [61]. This GLP-1 signaling-mediated increase in circulating ANP and brain natriuretic peptide (BNP) levels has been corroborated in human studies [62]. However, the GLP-1–ANP axis appears to be species-specific, with robust evidence currently limited to murine models [63].

### 3.2. Oxidative Stress and Vascular Inflammation Attenuation

In individuals with diabetic cardiomyopathy (DCM), aberrant glucose metabolism and insulin resistance may directly precipitate myocardial microvascular injury, distinguishing them from those with normal metabolic function [41,64]. Among the key pathological drivers, oxidative stress inflicts endothelial damage, disrupts NO-mediated vasodilation, and promotes the upregulation of vasoconstrictive factors, thereby impairing vascular homeostasis [65]. Beyond oxidative damage, localized vascular inflammation plays a pivotal role in endothelial dysfunction and compromised vasodilation [65]. Hyperglycemia and dyslipidemia further exacerbate these vascular abnormalities by perturbing the intricate crosstalk between immune cells and endothelial metabolic processes [66]. Notably, insulin analogs have demonstrated the capacity to partially restore the myocardial perfusion abnormalities observed in the postprandial state by improving glycemic control [67]. However, the therapeutic potential of GLP-1 and its receptor agonists extends beyond their role in modulating systemic glucose metabolism and insulin sensitivity. These agents confer direct endothelial protection and preserve myocardial perfusion by attenuating oxidative stress and mitigating vascular inflammation, underscoring their potential as multifaceted interventions in diabetic cardiovascular disease.

These cardioprotective effects of GLP-1 signaling appear to extend beyond its well-characterized metabolic regulatory functions [68,69]. Advanced imaging studies employing ^13^C-magnetic resonance spectroscopy (MRS) in animal models have revealed that GLP-1 activation markedly enhances myocardial glucose uptake and oxidative phosphorylation, thereby optimizing cellular energy metabolism [70]. In the streptozotocin (STZ)-induced diabetic rat model, treatment with vildagliptin or exenatide significantly increased cardiac microvascular density and reinforced endothelial barrier integrity, as evidenced by a reduction in lanthanum nitrate diffusion across endothelial cells [71]. Notably, this endothelial protection was independent of glucose-lowering effects, suggesting an alternative mechanistic pathway. A potential explanation lies in the inhibition of the Rho/ROCK signaling axis, a key mediator of oxidative stress-induced endothelial damage [71]. Further corroborating these findings, liraglutide has demonstrated profound vascular benefits in hypertensive mice [55]. Its administration led to substantial reductions in blood pressure, alleviated cardiac hypertrophy, mitigated vascular fibrosis, and improved endothelial function. Mechanistically, these effects were mediated through suppression of oxidative stress and vascular inflammation via GLP-1R activation. Specifically, liraglutide reduced NADPH oxidase activity, lowered asymmetric dimethylarginine (ADMA) levels, and decreased F4/80 expression in cardiac tissue, collectively reinforcing its protective role in maintaining coronary microvascular barrier integrity [55].

To elucidate the molecular mechanisms underlying its vascular protective effects, in vitro studies using coronary microvascular endothelial cells (CMECs) cultured under hyperglycemic conditions have demonstrated that GLP-1 mitigates high glucose-induced oxidative stress and apoptosis [55]. This protective effect is mediated through inhibition of the Rho/ROCK signaling cascade, a critical regulator of endothelial dysfunction and cytoskeletal remodeling [55]. Consistent with these findings, chronic liraglutide therapy in obese Zucker rats has been shown to enhance vasodilatory capacity across both the coronary macro- and microcirculation. This improvement is partially attributed to a 40% reduction in pro-inflammatory mediators, including NF-κB, CD68, IL-1β, and TGF-β1, within the myocardium [54]. Additionally, liraglutide influences macrophage polarization, a key determinant of inflammation resolution and tissue repair, further reinforcing its anti-inflammatory and cardioprotective properties [72]. Clinical studies in patients with T2D provide further support for these vascular benefits. The combination of GLP-1 with insulin has been associated with a significant reduction in systemic markers of vascular inflammation, including soluble intercellular adhesion molecule (sICAM-1), plasma 8-iso-prostaglandin F2α (8-iso-PGF2α), nitrotyrosine, and IL-6 [73]. By attenuating these inflammatory pathways, GLP-1 enhances endothelial function and restores vascular perfusion, underscoring its potential as a therapeutic strategy for improving microvascular integrity in metabolic disorders [73].

A recent study by Stone et al. [74] investigated the cardioprotective effects of semaglutide in a large animal model of coronary artery disease (CAD), independent of diabetes or obesity. Using a porcine model with induced myocardial ischemia, the study demonstrated that semaglutide administration significantly improved myocardial perfusion and left ventricular systolic performance [74]. These effects were accompanied by reduced perivascular and interstitial fibrosis, lower apoptotic activity, and enhanced activation of the AMPK–eNOS pathway [74]. Notably, these benefits were observed both at rest and during cardiac stress, highlighting the potential of GLP-1R agonists to enhance coronary microcirculation and myocardial adaptation to ischemia [74]. These findings suggest that GLP-1R activation may offer direct vascular benefits in CAD, beyond its metabolic effects, reinforcing its potential as an adjunctive therapy for ischemic heart disease [74].

### 3.3. Angiogenesis Stimulation

Angiogenesis plays a crucial role in patients for whom conventional revascularization strategies are not viable, as it facilitates microvascular expansion and enhances endothelial exchange capacity. Endothelial cells possess an intrinsic proliferative potential, enabling their active participation in neovascularization. GLP-1 has been shown to potentiate angiogenic signaling via the PI3K/Akt, PKA, and Src pathways, as demonstrated in HUVEC models [75]. Furthermore, vildagliptin-induced elevations in GLP-1 levels have been linked to the suppression of oxidative stress-mediated proteasome activation, thereby preventing the degradation of hypoxia-inducible factor-1α (HIF-1α). This stabilization of HIF-1α enhances VEGF activation, leading to increased capillary density and improved microvascular function [76]. Additional evidence highlights the angiogenic capacity of exendin-4, which has been shown to promote proliferation in human coronary artery endothelial cells (HCAECs) via PKA–PI3K/Akt–eNOS signaling [77]. Importantly, these angiogenic properties of GLP-1 signaling extend beyond in vitro models. In preclinical studies, sitagliptin administration to mice with critical limb ischemia resulted in elevated circulating GLP-1 and endothelial progenitor cell levels, ultimately enhancing neovascularization through an eNOS-dependent mechanism [78]. Table 1 provides an overview of key studies elucidating the mechanisms by which GLP-1 and its receptor agonists contribute to the improvement of coronary microvascular perfusion.

## 4. Clinical Evidence Supporting GLP-1 Receptor Agonists in Enhancing Myocardial Perfusion

Current clinical management of symptomatic coronary artery obstructions primarily involves percutaneous coronary intervention (PCI) or coronary artery bypass grafting (CABG), supplemented by pharmacotherapy with angiotensin converting enzyme (ACE) inhibitors, angiotensin II receptor blockers (ARBs), and antiplatelet agents for secondary prevention [80]. However, for coronary microvascular dysfunction (CMD), effective targeted therapies remain elusive. Existing interventions, including β-blockers, calcium channel blockers, and nitrates, offer limited efficacy and are often accompanied by adverse effects such as bradycardia, hypotension, peripheral edema, and drug tolerance. While PCI rapidly restores epicardial blood flow, its impact on microcirculation is minimal, and it carries procedural risks [81]. CABG, though effective for multi-vessel disease, is invasive and entails prolonged recovery [82]. Given these limitations, the need for novel pharmacological strategies to enhance coronary microvascular function is paramount. Given these challenges, there is a critical need for novel pharmacological interventions that specifically target the coronary microcirculation. Among the emerging therapeutic candidates, GLP-1R agonists have garnered considerable attention due to their potential to enhance endothelial function, improve microvascular perfusion, and mitigate CMD, positioning them as promising agents in the evolving landscape of cardiovascular therapeutics.

### Effect on Coronary Microcirculation

Both GLP-1 and GLP-1R agonists, whether administered as monotherapy or in combination with other agents, have demonstrated the capacity to enhance coronary microvascular dilation and augment microvascular blood volume (MBV). Under physiological conditions, GLP-1 infusion significantly increased myocardial MBV and microvascular blood flow (MBF) at both 30 and 150 min post-administration, while microvascular flow velocity (MFV) exhibited a slight reduction, indicating a redistribution of perfusion dynamics without compromising overall myocardial circulation [83].

Consistent with these findings, GLP-1 infusion alone has been shown to elicit a 40% increase in MBV within cardiac muscle, with no significant alteration in MFV, leading to a substantial enhancement in MBF [84]. Notably, this effect remains preserved in the myocardial microvasculature even in the context of obesity, underscoring its potential for broader cardiometabolic applications [84]. Additionally, exenatide has been observed to increase MBF independently of myocardial glucose uptake, suggesting a direct vasodilatory effect on the coronary microcirculation beyond its metabolic actions [85].

In conditions of restricted coronary perfusion, even a modest increase in MBV can significantly enhance tissue oxygenation and myocardial function. Evidence suggests that GLP-1 promotes coronary microvascular dilation and increased myocardial perfusion without altering peripheral vascular tone [86]. This selective vasodilatory effect may be attributed to GLP-1R activation on ventricular myocytes, which enhances myocardial contractility and induces secondary vasodilation within the coronary microcirculation [86].

A key measure of coronary endothelial function, coronary flow velocity reserve (CFVR) was significantly improved following 12 weeks of exenatide therapy, coinciding with a marked reduction in soluble intercellular adhesion molecule-1 (sICAM-1) and vascular cell adhesion molecule-1 (VCAM-1) levels, reflecting reduced endothelial inflammation [53]. Furthermore, while direct evidence of liraglutide’s effects on the coronary microcirculation remains limited, randomized controlled trials have demonstrated its ability to enhance endothelial function [55,87]. These benefits were characterized by upregulated eNOS expression, increased NO bioavailability, and reduced serum endothelin-1 (ET-1) and inflammatory markers, collectively indicating liraglutide’s potential to improve vascular homeostasis and modulate coronary microcirculatory dynamics [55,87].

Further reinforcing these findings, a randomized, open-label, crossover trial by Chowdhary et al. [14] investigated the effects of liraglutide on myocardial perfusion, cardiac energetics, and exercise capacity in patients with T2D but without established cardiovascular disease. Forty-one participants were randomized to receive either liraglutide or pioglitazone for 16 weeks, followed by an 8-week washout period, after which they switched to the alternate treatment [14]. Key findings revealed that liraglutide significantly enhanced myocardial perfusion, with a notable increase in stress MBF (1.62 to 2.08 mL/g/min, *p* = 0.01) and myocardial perfusion reserve (2.40 to 2.90, *p* = 0.01) [14]. Additionally, liraglutide improved myocardial energetics as evidenced by higher phosphocreatine-to-ATP ratios at rest and during stress [14]. Of note, these benefits translated into greater exercise capacity, demonstrated by a significant increase in the 6 min walk distance [14]. Another recent study assessed the effects of GLP-1R agonists on myocardial perfusion in obese individuals with high and very high cardiovascular risk using 99mTc-MIBI SPECT imaging [88]. After six months of treatment, myocardial perfusion inhomogeneity parameters significantly improved, particularly in patients with T2D or impaired glucose tolerance [88]. Notable reductions were observed in the stress impairment severity index and stress heterogeneity index, indicating enhanced coronary microvascular function [88].

In summary, GLP-1 and GLP-1R agonists play a protective role in the myocardium by reducing oxygen demand and enhancing coronary microcirculation through endothelial function improvements. Their non-invasive nature and favorable safety profile may further support greater patient adherence compared to conventional treatments. However, findings on their effects remain inconsistent, with some studies reporting no significant impact on coronary microcirculation [89,90,91]. A randomized, double-blind sub-study of the LIVE trial investigated the effects of liraglutide on myocardial glucose uptake (MGU), MBF, and myocardial flow reserve (MFR) in non-diabetic patients with stable chronic heart failure [89]. Over 24 weeks, liraglutide significantly reduced body weight and improved glycemic parameters, including HbA1c and 2 h post-OGTT glucose levels. However, no significant differences were observed between the liraglutide and placebo groups in terms of changes in MGU, MBF, or MFR, suggesting that liraglutide does not enhance myocardial perfusion or glucose metabolism in this patient population [89].

Another study by Faber et al. evaluated the short-term effects of liraglutide on coronary and peripheral microvascular function in patients with T2D without overt cardiovascular disease [90]. Over a 10-week treatment period, liraglutide led to a modest but non-significant increase in coronary flow reserve and had no measurable effect on peripheral endothelial function, assessed via reactive hyperemia index [90]. Of note, a randomized, double-blind, crossover study by Nilsson et al. investigated the acute effects of DPP-4 (increasing the level of intact GLP-1) (7–36) on coronary microvascular and peripheral endothelial function in overweight adults without diabetes [91]. Twelve participants received intravenous GLP-1 (7–36) or saline on separate occasions, alongside DPP-4 inhibition. Coronary flow velocity reserve and flow-mediated dilation were assessed as surrogate markers of vascular function [91]. The study found no significant effect of GLP-1 infusion on CFVR or FMD compared to saline, suggesting no direct short-term effect of intact GLP-1 on coronary or peripheral microvascular function in this population [91]. Collectively, these discrepancies may stem from small sample sizes and variations in study populations, particularly differences in baseline microvascular function (Table 2). To better define their role in coronary microvascular regulation, further well-designed, large-scale clinical trials are warranted.

## 5. Conclusions and Future Perspectives

The cardiovascular benefits of GLP-1R agonists extend beyond glucose regulation, with increasing evidence highlighting their role in enhancing coronary microvascular function and myocardial perfusion. Through mechanisms involving endothelial function preservation, NO bioavailability enhancement, oxidative stress reduction, and inflammatory pathway modulation, GLP-1R agonists exhibit vasoprotective effects that may mitigate CMD. Preclinical and clinical studies have demonstrated improvements in microvascular blood flow, myocardial perfusion reserve, and coronary endothelial function, particularly in patients with T2D and metabolic syndrome. Additionally, their angiogenic and cardioprotective properties suggest a potential role as adjunctive therapies in CAD. Despite these promising findings, heterogeneity in study designs, small sample sizes, and population-specific responses contribute to inconsistencies in reported benefits.

Notably, considerable variability exists among studies in terms of patient characteristics, imaging modalities, and definitions of coronary microvascular dysfunction, which may account for some of the inconsistencies observed in the reported outcomes. Differences in baseline cardiovascular risk, glycemic control, comorbidities, and methodological approaches—ranging from cardiac MRI and PET to Doppler-based assessments—pose challenges in directly comparing results across studies. Nonetheless, these diverse investigations consistently report favorable effects of GLP-1 receptor agonists on myocardial perfusion, suggesting that the observed benefits may be robust across a range of clinical contexts. In this light, the reproducibility of mainly positive findings despite methodological heterogeneity may itself support the broad therapeutic potential of GLP-1RAs in improving coronary microvascular function. Acknowledging these limitations, future research should prioritize large-scale, multicenter clinical trials to confirm the long-term cardiovascular impact of GLP-1R activation in diverse patient populations. Advanced imaging techniques such as myocardial contrast echocardiography, cardiac MRI, and PET scans should be employed to provide a more precise evaluation of GLP-1R agonists’ effects on coronary microcirculation. Further mechanistic studies are needed to elucidate the role of GLP-1R signaling in endothelial function, angiogenesis, and vascular remodeling beyond metabolic control. Additionally, exploring combination therapies involving GLP-1 receptor agonists and other cardioprotective agents—such as sodium–glucose co-transporter-2 (SGLT2) inhibitors, which have shown conflicting results regarding their impact on myocardial perfusion indices [93,94]—as well as established vasodilators, may offer synergistic benefits and enhance treatment efficacy in the management of coronary microvascular dysfunction. Personalized medicine approaches should also be explored, identifying patient subgroups that may derive the greatest benefit from GLP-1R-based therapies based on metabolic, genetic, or inflammatory biomarkers.

As research progresses, GLP-1R agonists may become integral to the management of CMD and ischemic heart disease, bridging the gap between metabolic control and cardiovascular protection and offering a novel, targeted therapeutic approach for patients with impaired coronary microvascular perfusion.

## Figures and Tables

**Table 1:** Mechanistic pathways through which GLP-1 and its agonists enhance coronary microvascular perfusion.

Study (Year)	Model	Intervention	Dosing	Mechanisms
Helmstädter et al. (2020) [55]	Mouse model of angiotensin II-induced arterial hypertension	Liraglutide	N/R	Reduced vascular inflammation, oxidative stress, and endothelial dysfunction via endothelial GLP-1R activation. Prevented eNOS uncoupling, increased NO bioavailability, reduced leukocyte rolling and infiltration, and decreased expression of vascular adhesion molecules (VCAM-1, ICAM-1, P-selectin).
Kim & Platt et al. (2013) [61]	Mouse model of angiotensin II-induced hypertension	Liraglutide	30 Βµg/kg intraperitoneally, twice daily for 3 weeks	GLP-1R activation in atrial cardiomyocytes increased atrial natriuretic peptide (ANP) secretion, which in turn reduced blood pressure via natriuresis and vasodilation. The mechanism involved Epac2-mediated ANP secretion, activation of natriuretic peptide receptor A, and increased cGMP signaling in vascular smooth muscle cells.
Kelly et al. (2012) [79]	Human (obese, pre-diabetic patients)	Exenatide	5 mcg BID for 1 month, then 10 mcg BID for 2 months	No significant improvement in microvascular endothelial function (RHI), inflammation (CRP), oxidative stress (oxLDL), or vascular activation (VCAM-1) compared to metformin. However, exenatide significantly reduced triglycerides, which may have postprandial vascular benefits.
Ceriello et al. (2014) [73]	Human (patients with type 2 diabetes)	GLP-1 infusion with or without insulin	0.4 pmol/kg/min infusion during 2 h glucose clamps	GLP-1 improved flow-mediated dilation (FMD), reduced markers of inflammation (IL-6, sICAM-1), and oxidative stress (8-iso-PGF2a, nitrotyrosine). Effects were enhanced when GLP-1 was combined with insulin, suggesting a synergistic vasodilatory, anti-inflammatory, and antioxidant action.
Stone et al. (2025) [74]	Large animal model (Yorkshire swine with coronary artery disease)	semaglutide	Oral 1.5 mg, increased to 3 mg over 2 weeks, continued for 5 weeks	Improved myocardial perfusion and systolic function through activation of the AMPK-eNOS pathway, leading to enhanced endothelial function and coronary vasodilation. Reduced perivascular fibrosis, interstitial fibrosis, and apoptosis, suggesting an additional role in myocardial remodeling and cellular survival.

Abbreviations: N/R, not reported.

**Table 2:** Impact of GLP-1 receptor agonists on coronary microvascular dynamics and function.

Study (Year)	Population	Number of Participants	Imaging Modality	Results	Implications
Subaran et al. (2014) [83]	Healthy adults (18–35 years)	26	Contrast-enhanced ultrasound (CEU)/Myocardial contrast echocardiography (MCE)	GLP-1 infusion significantly increased myocardial microvascular blood volume (MBV) by ~53% at 30 min and ~57% at 150 min. Myocardial blood flow (MBF) increased by ~48% at 30 min and ~47% at 150 min. Microvascular flow velocity (MFV) slightly decreased.	GLP-1 receptor activation enhances myocardial microvascular recruitment, improving tissue oxygen and nutrient delivery. This suggests a potential role for GLP-1 receptor agonists in preserving coronary microvascular function.
Chowdhary et al. (2024) [14]	Patients with T2D without established cardiovascular disease	41	CMR and 31-phosphorus magnetic resonance spectroscopy (31P-MRS)	Liraglutide significantly improved stress myocardial blood flow (1.62 to 2.08 mL/g/min, *p* = 0.01) and myocardial perfusion reserve (2.40 to 2.90, *p* = 0.01). Rest and stress phosphocreatine-to-ATP ratios increased, indicating enhanced myocardial energetics.	GLP-1 receptor agonist liraglutide enhances myocardial perfusion and energetics, supporting its therapeutic potential in patients with T2D at risk of microvascular dysfunction.
Nilsson et al. (2019) [91]	Obese adults without diabetes	12	Trans-thoracic Doppler echocardiography	No significant difference in coronary flow velocity reserve (CFVR) between GLP-1 infusion (3.77 ± 1.25) and saline infusion (3.85 ± 1.32). No significant effect on peripheral endothelial function.	Acute GLP-1 infusion did not improve coronary microcirculation in obese, glucose-tolerant adults, suggesting its effects may depend on metabolic status or require long-term treatment.
Clarke et al. (2018) [86]	Patients with stable angina awaiting LAD stenting	21	Pressure-flow wire assessment of coronary blood flow	GLP-1 reduced resting coronary transit time (0.87 to 0.63 s, *p* = 0.02) and basal microcirculatory resistance (76.3 to 55.4 mmHg/s, *p* = 0.02), whereas controls exhibited an increase in both parameters. No significant effect on systemic hemodynamics or peripheral vascular tone.	GLP-1 promotes coronary microvascular dilation and enhances myocardial blood flow through ventricular-coronary crosstalk, suggesting a potential cardioprotective role independent of systemic vasodilation.
Aetesam-Ur-Rahman et al. (2021) [92]	Patients undergoing PCI for stable angina	41	Pressure wire assessment of coronary distal pressure and flow velocity (thermodilution transit time—Tmn)	GLP-1 caused a significant reduction in resting Tmn and basal microvascular resistance (BMR), indicating improved coronary microvascular function. The vasodilatory effect was not attenuated by theophylline, suggesting an adenosine-independent mechanism.	GLP-1 receptor activation improves coronary microvascular function via an adenosine-independent pathway, supporting its potential role in microvascular dysfunction management. Further research is needed to elucidate alternative mechanisms.
Chen et al. (2016) [87]	Patients with STEMI undergoing primary PCI	92	Transthoracic echocardiography	Liraglutide significantly improved left ventricular ejection fraction (LVEF) at 3 months compared to placebo (+4.1%, 95% CI: +1.1% to +6.9%, *p* < 0.001). Reduction in inflammatory markers and endothelial dysfunction indicators was observed.	Short-term liraglutide therapy post-STEMI may support myocardial recovery by enhancing left ventricular function and reducing endothelial inflammation, warranting larger-scale trials.
Faber et al. (2015) [90]	Patients with T2D and no coronary artery disease history	24	Trans-thoracic Doppler-flow echocardiography	Liraglutide led to a small, non-significant increase in coronary flow reserve (CFR) (change: 0.18, 95% CI: [−0.01, 0.36], *p* = 0.06). No significant difference in CFR between liraglutide and control (difference: 0.16, 95% CI: [−0.08, 0.40], *p* = 0.18).	Short-term liraglutide treatment did not significantly enhance coronary microvascular function. Future studies should explore long-term effects and higher dosing in patients with greater microvascular impairment.
Gejl et al. (2012) [85]	Insulin-naive male patients with T2D without coronary artery disease	8	Positron emission tomography (PET) with 18F-fluorodeoxyglucose and 13N-ammonia	Exenatide increased myocardial blood flow (MBF) by 24% (0.69 ± 0.097 to 0.86 ± 0.09 mL/g/min, *p* = 0.0089), but had no effect on myocardial glucose uptake (MGU).	GLP-1 receptor activation with exenatide enhances myocardial perfusion without altering glucose uptake, suggesting a potential vasodilatory effect on coronary microcirculation in patients with T2D. Further research is needed to explore its long-term benefits.
Nielsen et al. (2019) [89]	Patients with stable chronic heart failure and reduced ejection fraction (≤45%)	36	Positron emission tomography (PET) with 18F-FDG and 15O-H_2_O	Liraglutide treatment for 24 weeks had no significant effect on myocardial glucose uptake (MGU), myocardial blood flow (MBF), or myocardial flow reserve (MFR) compared to placebo (*p* = 0.98, *p* = 0.76, and *p* = 0.89, respectively).	Liraglutide does not enhance myocardial perfusion or glucose metabolism in non-diabetic patients with heart failure. The absence of effects on myocardial perfusion may explain the lack of observed cardiovascular benefit in heart failure trials involving GLP-1 receptor agonists.
Wei et al. (2016) [53]	Patients with newly diagnosed T2D	36	Transthoracic Doppler echocardiography	Exenatide significantly improved coronary flow velocity reserve (CFVR) (baseline: 2.89 ± 0.60, post-treatment: 3.36 ± 0.58, *p* < 0.05). Significant reduction in inflammatory markers sICAM-1 and sVCAM-1 post-treatment.	Exenatide enhances coronary endothelial function and reduces vascular inflammation in newly diagnosed T2D patients, suggesting a potential role in mitigating cardiovascular risk.
Rezinkina et al. (2023) [88]	Obese patients with high and very high cardiovascular risk	30 (15 with T2D/IGT, 15 without carbohydrate metabolism disorders)	99mTc-MIBI SPECT (rest/stress)	After 6 months of GLP-1 receptor agonist therapy, myocardial perfusion inhomogeneity significantly improved, with reductions in stress σsev (26.8 ± 5.7 to 22.6 ± 4.7, *p* = 0.03) and stress σhet (10.6 ± 3.1 to 9.1 ± 2.5, *p* = 0.061). Improvements were more pronounced in the T2D/IGT group.	GLP-1 receptor agonists improve myocardial perfusion at the microcirculatory level in high-risk obese patients, particularly those with T2D/IGT. Further research is needed to refine patient selection and optimize cardiovascular outcome assessments.

Abbreviations: ATP (Adenosine Triphosphate), BMR (Basal Microvascular Resistance), CFR (Coronary Flow Reserve), CFVR (Coronary Flow Velocity Reserve), CMR (Cardiovascular Magnetic Resonance), FDG (Fluorodeoxyglucose), GLP-1 (Glucagon-Like Peptide-1), IGT (Impaired Glucose Tolerance), LAD (Left Anterior Descending Artery), LVEF (Left Ventricular Ejection Fraction), MBF (Myocardial Blood Flow), MBV (Microvascular Blood Volume), MCE (Myocardial Contrast Echocardiography), MFV (Microvascular Flow Velocity), MFR (Myocardial Flow Reserve), MGU (Myocardial Glucose Uptake), MIBI (Methoxyisobutylisonitrile), PCI (Percutaneous Coronary Intervention), PET (Positron Emission Tomography), STEMI (ST-Elevation Myocardial Infarction), sICAM-1 (Soluble Intercellular Adhesion Molecule-1), sVCAM-1 (Soluble Vascular Cell Adhesion Molecule-1), and T2D (Type 2 Diabetes).

## Data Availability

All data generated in this research is included within the article.
